# The San1 Ubiquitin Ligase Avidly Recognizes Misfolded Proteins through Multiple Substrate Binding Sites

**DOI:** 10.3390/biom11111619

**Published:** 2021-11-02

**Authors:** Rebeca Ibarra, Heather R. Borror, Bryce Hart, Richard G. Gardner, Gary Kleiger

**Affiliations:** 1 Department of Chemistry and Biochemistry, University of Nevada, Las Vegas, NV 89154, USA; ibarra10@unlv.nevada.edu (R.I.); hartb1@live.unc.edu (B.H.); 2 Department of Pharmacology, University of Washington, Seattle, WA 98195, USA; hborror@uw.edu (H.R.B.); gardnerr@uw.edu (R.G.G.)

**Keywords:** protein quality control, ubiquitylation, protein degradation, ubiquitin ligase

## Abstract

Cellular homeostasis depends on robust protein quality control (PQC) pathways that discern misfolded proteins from functional ones in the cell. One major branch of PQC involves the controlled degradation of misfolded proteins by the ubiquitin-proteasome system. Here ubiquitin ligases must recognize and bind to misfolded proteins with sufficient energy to form a complex and with an adequate half-life to achieve poly-ubiquitin chain formation, the signal for protein degradation, prior to its dissociation from the ligase. It is not well understood how PQC ubiquitin ligases accomplish these tasks. Employing a fully reconstituted enzyme and substrate system to perform quantitative biochemical experiments, we demonstrate that the yeast PQC ubiquitin ligase San1 contains multiple substrate binding sites along its polypeptide chain that appear to display specificity for unique misfolded proteins. The results are consistent with a model where these substrate binding sites enable San1 to bind to misfolded substrates avidly, resulting in high affinity ubiquitin ligase-substrate complexes.

## 1. Introduction

All living cells are burdened with the task of disposing of misfolded or damaged proteins [1]. Termed protein quality control (PQC) [2,3,4,5,6,7], these molecular pathways guard against the accumulation of misfolded proteins in cells, which if left unchecked, may result in the malfunction of normal cellular processes. PQC is especially relevant to human biology where its breakdown is well known to play roles in the development of various disease states including neurodegeneration and cancer [8,9,10,11,12,13,14,15]. In eukaryotic cells, the ubiquitin system, a signal transduction cascade that targets proteins for degradation, is a major regulator of PQC [16,17,18,19,20]. Here poly-ubiquitin chains are formed onto misfolded proteins bound to ubiquitin ligases, enzymes that scrutinize misfolded proteins from normal ones [21]. Many mechanistic aspects regarding how PQC ubiquitin ligases function remain unresolved, such as how misfolded proteins are recognized, and how sufficient binding energy is achieved to stabilize ligase-substrate complexes that are held together presumably through weak, nonspecific hydrophobic interactions. Uncovering how ubiquitin ligases recognize misfolded protein substrates is an important step towards understanding the molecular pathologies of human diseases that are linked to aberrant PQC processes.

Addressing how ubiquitin ligases bind to and recognize misfolded proteins requires robust in vitro reconstituted systems that enable biochemical, biophysical, and structural biological inquiries into function. This is challenging work [22,23,24] owing to the nature of misfolded substrates and in at least some cases even the PQC-specific ubiquitin ligases themselves that collectively contain stretches of solvent-exposed hydrophobic residues that can lead to aggregation at even relatively low concentrations. Thus, most of the reconstituted systems in PQC to date require at least some of the components being from crude lysate instead of a highly purified source. Nevertheless, amazing progress has been achieved with fully reconstituted systems in related fields such as molecular chaperones [25] and inspired our campaign to find a PQC ubiquitin ligase amenable to in vitro biochemical experiments.

Amongst the best characterized PQC-specific ubiquitin ligases is San1, a *Saccharomyces cerevisiae* enzyme that recognizes and ubiquitylates misfolded proteins in the nucleus [26,27,28,29,30,31,32,33,34,35,36,37,38,39,40,41,42,43,44,45,46,47]. San1 has several characteristics that imply potential utility for in vitro biochemical experiments. San1 is a modestly sized protein (approximately 65 kDa), and despite it containing long stretches of disordered regions along the poly-peptide chain, San1 is not known to oligomerize [45]. A key breakthrough towards a fully reconstituted system was the development of a small peptide substrate that is ubiquitylated by San1 [37]. Unlike most misfolded proteins, the peptide substrate displays remarkable solubility, with no visual precipitation even at concentrations in the low millimolar range, and can be used reproducibly and quantitatively at up to 10 μM in kinetic assays. These properties enabled for the first time quantitative kinetic assays and new insights into San1′s molecular function. Perhaps most important is that an understanding of San1 molecular function from assays performed in yeast cells indicated novel modes of substrate recognition and suggested biochemical experiments to test these hypotheses.

In a landmark study, the *Saccharomyces cerevisiae* nuclear PQC ubiquitin ligase San1 was shown to contain multiple contiguous disordered regions in the primary structure that appear to bind to San1 protein substrates [45], leading to two distinct hypotheses. First, San1 substrate binding sites may each recognize misfolded protein substrates with little or even no substrate specificity. On the other hand, San1 substrate binding sites may display both sequence and structural specificities for substrate. To test these hypotheses, the utility of the reconstituted San1 PQC ubiquitylation reaction system was significantly improved by identifying a San1 truncation mutant that is far more amenable to biochemical approaches and yet recapitulates the in vitro activities of full-length San1. We then demonstrate that aspects of both hypotheses for San1 substrate binding appear to be valid. While San1 harbors multiple substrate binding sites that seem to recognize distinct substrates, the evidence also supports the notion that at least some binding sites also bind to the same substrates. Remarkably, these sites endow San1 with the ability to bind to substrates with high affinity, suggesting that substrate binding to San1 is driven by an avidity between substrates and San1′s multiple substrate binding regions.

## 2. Materials and Methods

### 2.1. Expression and Purification of Recombinant Proteins

Since wild-type San1 protein has been shown to rapidly auto-ubiquitylate, all experiments performed for these studies were with San1 constructs where lysine residues had been changed to arginines. Full-length San1 was purified as previously described [37]. San1_1–303_ was purified similarly with a few notable modifications. Briefly, proteins were expressed in *Escherichia coli* using Rosetta 2(DE3)pLysS competent cells (Novagen; Gibbstown, NJ, USA). Bacterial cells were grown at 37 °C to an optical density of 0.8–1.0, after which expression was induced with 0.1 mM IPTG for 2 h followed by centrifugation and storage of the cell pellets at −80 °C. Bacterial cell pellets were solubilized in lysis buffer containing 30 mM Tris, pH 7.5, 200 mM NaCl, 5 mM DTT, 1 mM EDTA, 10% glycerol, and protease inhibitor cocktail (Thermo; Waltham, MA, USA) and disrupted by multiple rounds of sonication. Lysates were prepared by centrifugation and then incubated with Glutathione Sepharose 4B beads (GE Healthcare Life Sciences; Chicago, IL, USA) for 3 h at 4 °C. Beads were then collected and washed repeatedly with lysis buffer lacking protease inhibitors and EDTA. Recombinant GST- San1_1–303_ protein was eluted in a buffer containing 50 mM tris, pH 8.0, 200 mM NaCl, and 40 mM glutathione. The protein was then incubated with TEV protease overnight at 4 °C, followed by loading onto a 1 mL HisTrap HP column (GE Healthcare Life Sciences; Chicago, IL, USA) that had been equilibrated in buffer A containing 50 mM HEPES, pH 7.5, 200 mM NaCl, 20 mM imidazole, and 5% glycerol. Histidine-tagged San1 proteins were eluted from the column using a linear gradient of buffer B containing 50 mM HEPES, pH 7.5, 200 mM NaCl, 300 mM imidazole, and 5% glycerol. Fractions containing San1_1–303_ were concentrated and then injected onto a Superdex 200 gel filtration column (GE Healthcare; Chicago, IL, USA). Fractions containing San1_1–303_ were concentrated (Amicon Ultra-4 10,000 NMWL; Burlington, MA, USA) to approximately 10 µM and flash frozen in liquid nitrogen prior to storage at −80 °C.

Human E1 and Ubc1p were purified as previously described [37]. Recombinant ubiquitin was purchased from Boston Biochem. Peptide substrates were purchased from New England Peptide. The peptide amino acid sequences are as shown and with acetylated N-termini.

- San1 peptide  N-CGSRRGSYNASSGEQMLSRTGFFLVLIVGQPLHNPVK-Cterm
- KR San1 peptide N-CGSRRGSYNASSGEQMLSRTGFFLVLIVGQPLHNPVR-Cterm

### 2.2. Limited Proteolysis

San1 chymotrypsin digestion assays were performed in a buffer containing 30 mM Tris, pH 7.5, 100 mM CaCl_2_, and 2 mM DTT. All reactions contained 0.25 µM radiolabeled full-length San1 or San1_1–303_ and were supplemented with 0.1% tween-20. Reactions were then incubated at room temperature in either the absence or presence of 5 µM San1 peptide for two minutes followed by the addition of a 1:100 molar ratio of chymotrypsin (Sigma-Aldrich; St. Louis, MO, USA). Time-points were quenched in 2X SDS-PAGE and boiled for 5 min at 95 °C. Substrate and products were resolved by SDS-PAGE on 4–20% gels, dried, and exposed on a phosphor screen. Autoradiography was performed using a Typhoon 9410 imager. Quantification of substrate and products were performed with ImageQuant software (GE Healthcare; Chicago, IL, USA). San1-trypsin digestion assays were performed similarly except in a buffer containing 30 mM Tris, pH 7.5, 5 mm MgCl_2_, 2 mM ATP, 2 mM DTT, and 0.1% Tween-20 and with a 1:20 molar ratio trypsin to San1. Reactions with Firefly Luciferase (Sigma-Aldrich; St. Louis, MO, USA) and trypsin were performed similarly as above except all steps were performed at 42 °C prior to quenching.

### 2.3. Multi-Turnover Ubiquitylation Reactions

The San1 peptide was radiolabeled (50 µM) in the presence of γ-^32^P labeled ATP (Perkin Elmer; Waltham, MA, USA) and cAMP-dependent Protein Kinase (New England Biolabs; Ipswich, MA, USA) for 1 h at 30 °C in a reaction buffer that had been supplemented with tween-20 (0.1%). All reactions were performed in a buffer containing 30 mM Tris, pH 7.5, 5 mm MgCl_2_, 2 mM ATP, 2 mM DTT, and 0.1% Tween-20. Human E1 (1 µM), WT ubiquitin (60 µM), Ubc1 (10 µM), and either full-length San1 or San1_1–303_ (0.5 µM) were sequentially added to Eppendorf tubes and incubated for 2 min at room temperature. Next, 3 µM radiolabeled San1 peptide, 3 µM radiolabeled San1 peptide mixed with 3 µM unlabeled San1 peptide, or 3 µM radiolabeled San1 peptide mixed with 6 µM unlabeled San1 peptide were then added to initiate the respective ubiquitylation reactions. Reactions were quenched at various time-points in 2X SDS-PAGE buffer and substrate and ubiquitylated products were separated by SDS-PAGE on 4–20% gels (Lonza; Basel, Switzerland). Gels were dried and exposed to phosphor screens for autoradiography. The quantification of substrates and products was performed as described in the limited proteolysis section. The fraction of ubiquitylated San1 peptide was calculated by dividing the amount of peptide that had been modified by one or more ubiquitins by the total signal in the lane.

### 2.4. Single-Encounter Ubiquitylation Reactions

All single-encounter reactions were performed in a buffer containing 30 mM Tris, pH 7.5, 5 mm MgCl_2_, 2 mM ATP, 2 mM DTT, and 0.1% Tween-20. E1 (1 µM), WT human Ub (60 µM), and Ubc1 (10 µM) were incubated at room temperature to form activated ubiquitin-Ubc1 complex (tube 1). In a separate tube, full-length San1 or San1_1–303_ (1 µM) and labeled San1 Peptide (1 µM) were incubated to form a complex (tube 2). Ubiquitylation reactions were initiated by mixing tubes 1 and 2 together at room temperature. KR San1 peptide (10 µM) was added to either tube 1 or tube 2 as a negative control or for single-encounter ubiquitylation, respectively. Substrate and products were separated by SDS-PAGE on 4–20% gels, followed by processing and quantification as described in the multi-turnover ubiquitylation reactions section.

### 2.5. Nickel Pull-Down

For binding reactions containing peptide substrate, the San1 peptide was radiolabeled (50 µM) as described in the multi-turnover ubiquitylation reactions section. A total of 5 µM Radiolabeled San1 Peptide was then incubated with 0.1% tween and either 0.5 µM full-length San1 or KR San1_1–303_ for 5 min at room temperature. Binding reactions were diluted with 1 mL of nickel wash buffer containing 30 mM Tris, pH 7.5, 250 mM NaCl, 20 mM Imidazole, 0.1% Tween-20, and 5% Glycerol and incubated with 20 µL Nickel-NTA Agarose beads (Qiagen; Germantown, MD, USA) with gentle agitation for 1 h at room temperature. Reactions were then spun down at 1000× *g* for 2 min and 1 mL of additional wash buffer was introduced to the beads in the absence or presence of 5 µM unlabeled San1 peptide. Reactions were incubated for an additional 2 h under gentle agitation at room temperature. Beads were spun down and washed twice with wash buffer (containing no cold peptide). In total, 20 µL of 2X SDS Page buffer was added to the beads and boiled for 5 min at 95 °C. Bead-bound proteins were resolved by SDS-PAGE on 4–20% gels, dried, and exposed to a phosphor screen to perform autoradiography. The fraction of radiolabeled substrate bound to the beads was calculated as a fraction of the total input amount.

For binding reactions containing Firefly Luciferase (Sigma Aldrich; St. Louis, MO, USA), 0.5 µM luciferase was incubated with either 0.5 µM full-length San1 or KR San1_1–303_ for 5 min at 50 °C. Reactions were diluted with 1 mL of warmed nickel wash buffer and incubated with 20 µL Nickel-NTA Agarose beads with gentle agitation for 1 h at 50 °C. Reactions were then centrifuged at 3000× *g* for 30 s and washed three times with warmed nickel wash buffer. 20 µL of 2X SDS Page buffer was added to the beads and boiled for 5 min at 95 °C. Bead-bound products were transferred to nitrocellulose paper using a BioRad Semidry Transfer Cell Trans Blot SD and blocked in 5% nonfat milk in TBST for 1 h at room temperature. The membrane was then incubated with anti-luciferase antibody (Sigma Aldrich; St. Louis, MO, USA) in 0.5% milk using a 1:5000 dilution overnight at 4 °C. The secondary antibody that had been conjugated to Alexafluor 488 (Invitrogen; Waltham, MA, USA) was diluted 1:5000 in 0.1% milk and incubated with the membrane for 1 h at room temperature. Signal was detected using a Typhoon 9410 imager.

### 2.6. Luciferase Substrate Multi-Turnover Ubiquitylation Reactions

Reactions were performed in a buffer containing 30 mM Tris, pH 7.5, 5 mm MgCl_2_, 2 mM ATP, 2 mM DTT, and 0.1% Tween-20. E1 (1 µM), WT human Ub (60 µM), Ubc1 (10 µM), and either full-length or San1_1–303_ (0.5 µM) were incubated at room-temperature. In competition reactions, unlabeled KR San1 Peptide (10 µM) was added to the mixture and incubated for 2 min at 42 °C. Luciferase (0.5 µM) was then added to initiate the reactions that were then quenched with 2X SDS-PAGE loading buffer at the indicated time points. Substrate and product were resolved by SDS-PAGE on 4–20% gels. Substrates and products were transferred to nitrocellulose paper using a BioRad Semidry Transfer Cell Trans Blot SD and blocked in 5% milk in TBST buffer for 1 h at room temperature. The membrane was next incubated with anti-luciferase antibody (Sigma Aldrich; St. Louis, MO, USA) in 0.5% milk and TBST buffer at a 1:5000 dilution overnight at 4 °C. Secondary anti-rabbit antibody (Sigma Aldrich; St. Louis, MO, USA) diluted 1:5000 in 0.1% milk was incubated with the membrane for 1 h at room temperature. The membrane was imaged using Western Bright ECL (VWR; Radnor, PA, USA) on a BioRad ChemiDoc XRS+.

## 3. Results

**Figure 1:**
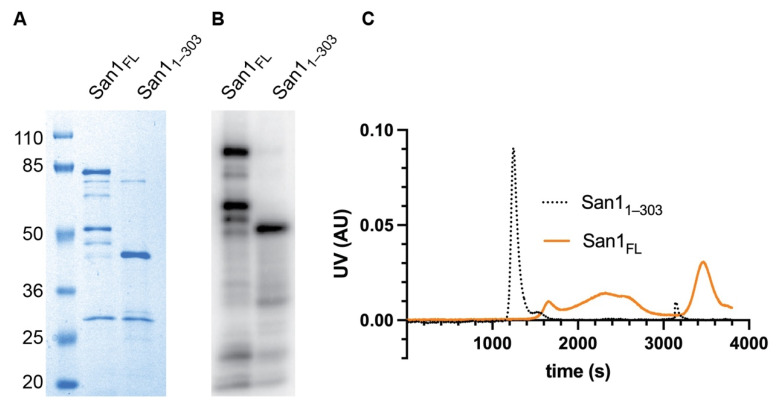
C-terminal truncation of San1 at the RING domain, San1_1–303_, results in greater purity and homogeneity compared with full-length. (**A**) Coomassie blue-stained SDS-PAGE gel showing the relative purities of full-length San1 versus San1_1–303_. Identical amounts of protein were loaded to each lane according to their optical densities at 280 nanometers. Notice that both proteins migrate significantly slower than their predicted molecular weights (full-length ~67.5 kDa and San1_1–303_ ~35 kDa), likely due to a lower content of hydrophobic residues. (**B**) The proteins in (**A**) were radiolabeled with ^32^P phosphate owing to the presence of a protein kinase A site at the N-terminus, followed by SDS-PAGE and detection by autoradiography. (**C**) Ultraviolet absorbance units (UV AU) comparing the migration of full-length San1 and San1_1–303_ by gel filtration chromatography.

We began our investigation by attempting to improve the reconstituted ubiquitylation system since full-length recombinant San1 protein is highly prone to proteolysis, resulting in degradation products occurring even after multiple rounds of purification and with affinity-based tags at both the N- and C-terminus (Figure 1A,B). Interestingly the major San1 degradation product contained an intact N-terminus (Figure 1B). Analysis of the San1 primary sequence suggested that the C-terminus of this fragment may be near the Really Interesting New Gene (RING) domain, which recruits activated ubiquitin to the San1-substrate complex. Thus, a construct was generated that expressed the original N-terminus of San1 and terminating shortly after the RING domain (1–303). This San1 protein, referred to as San1_1–303_ hereafter, migrated similarly by SDS-PAGE as the major degradation product of full-length San1 as expected. We reasoned that San1_1–303_ may retain similar levels of activity as full-length if San1 substrate binding sites are redundant.

The initial characterization of San1_1–303_ suggested that it may be more amenable to in vitro biochemistry than full-length San1. First, the expression level of San1_1–303_ in *Escherichia coli* was far higher in comparison with full-length San1, and the final level of purity was also significantly improved (Figure 1A,B). Secondly, while full-length San1 protein eluted in fractions from size exclusion chromatography spanning the entire run, San1_1–303_ eluted as a single peak, suggesting that it may represent a homogenous molecular species in contrast with the full-length protein (Figure 1C).

**Figure 2:**
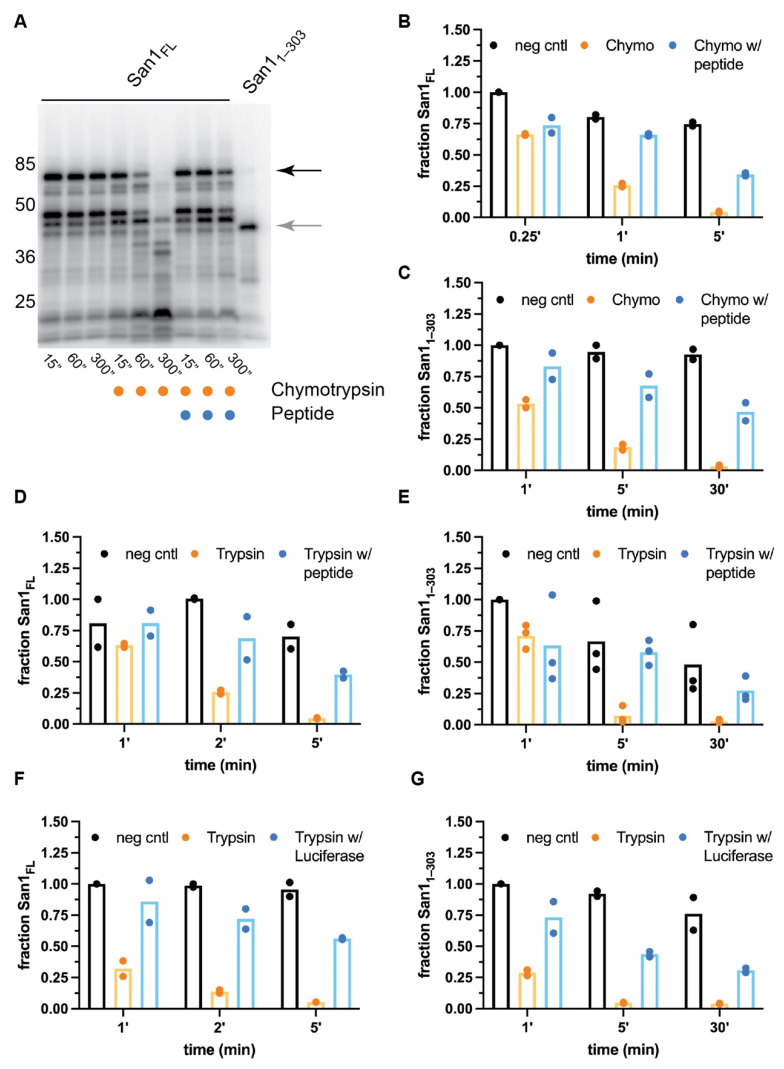
Peptide substrate significantly protects both full-length San1 and San1_1–303_ from proteolysis. (**A**) Representative autoradiogram of a time-course for untreated, radiolabeled full-length San1 or San1_1–303_ in the presence of chymotrypsin or in combination with peptide substrate. The migration of full-length San1 is noted by the black arrow on the right; San1_1–303_ is shown in the rightmost lane (gray arrow). (**B**) Graph of the results in (**A**) where the fraction of full-length San1 remaining was normalized to the 15” time-point for the untreated sample (leftmost lane). (**C**) same as (**B**), except with San1_1–303_. (**D**) same as (**B**), except with the protease trypsin. (**E**) Same as (**C**), except with the protease trypsin. (**F**) same as (**D**), except with heat-denatured luciferase as substrate. (**G**) same as (**E**), except with heat-denatured luciferase substrate. Representative autoradiograms for the graphs shown in panels (**B**) through (**G**) can be found in Figure S1A–E, respectively. The results all show duplicate data points from technical experimental replicates.

The biochemical properties of both full-length San1 as well as San1_1–303_ were next compared by performing limited proteolysis with chymotrypsin, a protease that recognizes aromatic and hydrophobic residues. Consistent with previous studies, full-length San1 protein was rapidly proteolyzed, likely owing to the multiple putative substrate binding sites that are disordered and contain residues recognized by chymotrypsin (Figure 2A). We hypothesized that if the sites of proteolysis overlap with substrate binding regions, the addition of substrate may protect San1 from proteolysis. A previously described San1 peptide substrate was employed owing to its unusually high solubility in comparison to misfolded proteins [37]. Remarkably, a 20-fold molar excess of peptide substrate over San1 resulted in substantial protection of full-length San1 protein in comparison with proteolysis reactions that lacked substrate (Figure 2A,B).

The stability of San1_1–303_ appeared to be slightly more resistant to chymotrypsin activity than full-length San1 (Figure 2C and Figure S1A), and the addition of excess peptide substrate also protected San1_1–303_ from proteolysis. Nearly 50% of San1_1–303_ remained intact in the presence of chymotrypsin after 30 min, resulting in an approximate 30-fold increase in the stability of San1_1–303_ protein in comparison with the absence of substrate. Since the peptide substrate contains residues that are recognized by chymotrypsin, it cannot be ruled out that at least some amount of San1 protection may be attributed to competition between San1 and excess peptide substrate for the protease active site. It is also intriguing to consider whether a chymotrypsin-resistant substrate may result in greater protection of San1. Similar results were obtained when both full-length San1 or San1_1–303_ were treated with trypsin (Figure 2D,E and Figure S1B,C). To assess whether a globular, misfolded protein substrate may protect San1 from proteolysis, heat-denatured luciferase (which had previously been shown to be ubiquitylated by San1 [37,45]) was added to San1 prior to its treatment with protease. Both full-length San1 and San1_1–303_ were significantly protected from trypsin-mediated proteolysis in the presence of misfolded luciferase (Figure 2F,G and Figure S1D,E). While luciferase, a 64 kDa protein, may be capable of protecting long stretches of San1 residues from proteolysis, the peptide substrate is only approximately 4 kDa, implying that multiple peptide molecules may be bound to both full-length San1 and San1_1–303_. In summary, these results support the notion that San1 contains multiple disordered substrate binding sites.

### 3.1. San1 Has Multiple High-Affinity Binding Sites for Substrate

**Figure 3:**
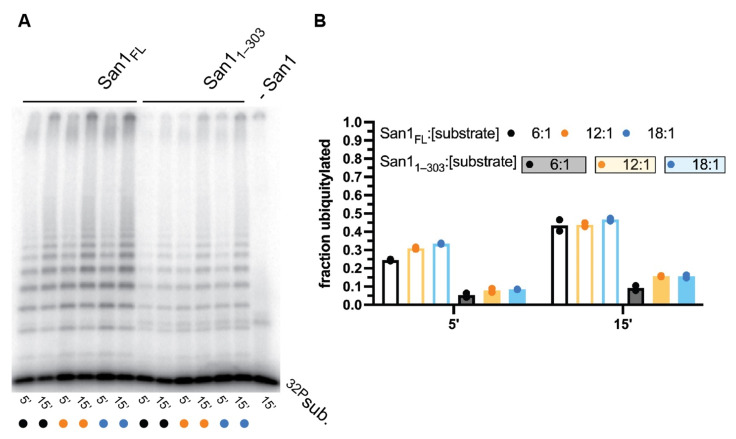
Similar fractions of peptide substrate are converted to ubiquitylated product by either full-length San1 or San1_1–303_ over a wide range of substrate levels. (**A**) Representative autoradiogram of multi-turnover ubiquitylation reactions containing substrate to San1 ratios of 6:1 (lanes 1–2 for full-length; lanes 7–8 for San1_1–303_), 12:1 (lanes 3–4 for full-length; lanes 9–10 for San1_1–303_), or 18:1 (lanes 5–6 for full-length; lanes 11–12 for San1_1–303_). The rightmost lane is a negative control reaction containing all necessary components for substrate ubiquitylation except San1. (**B**) Graph showing the fraction of substrate from (**A**) that had been converted to ubiquitylated products containing one or more ubiquitins. The results show duplicate data points from technical experimental replicates.

To explore whether peptide substrates can simultaneously bind to multiple sites along full-length San1 as well as San1_1–303_, multi-turnover kinetics were performed. For full-length San1, the fraction of substrate converted to ubiquitylated products was highly similar for all substrate to San1 ratios tested (Figure 3A,B). Some 30% of substrate had become ubiquitylated after 5 min, and nearly 50% after 15 min, even when substrate was in 18-fold molar excess of San1. These observations may reflect classical multi-turnover kinetics where the rapid dissociation of ubiquitylated products from San1 allows for additional rounds of substrate ubiquitylation during the time course. Alternatively, substrate and ubiquitylated products may bind tightly to San1 and the existence of additional unoccupied substrate binding sites would enable similar ratios of substrate conversion to product upon increasing substrate levels relative to San1.

Multi-turnover ubiquitylation assays were next performing with San1_1–303_ (Figure 3A,B). Similar to full-length San1, the fraction of substrate that had been converted to product was consistent for all ratios of substrate to San1_1–303_. However, the total fraction of substrate converted to product was approximately 3-fold lower than in comparison with full-length San1 (Figure 3B). The implications for these observations have been addressed in the Discussion section. In summary, these results are consistent with either rapid multi-turnover kinetics, the existence of multiple peptide substrate binding sites, or a combination of both.

**Figure 4:**
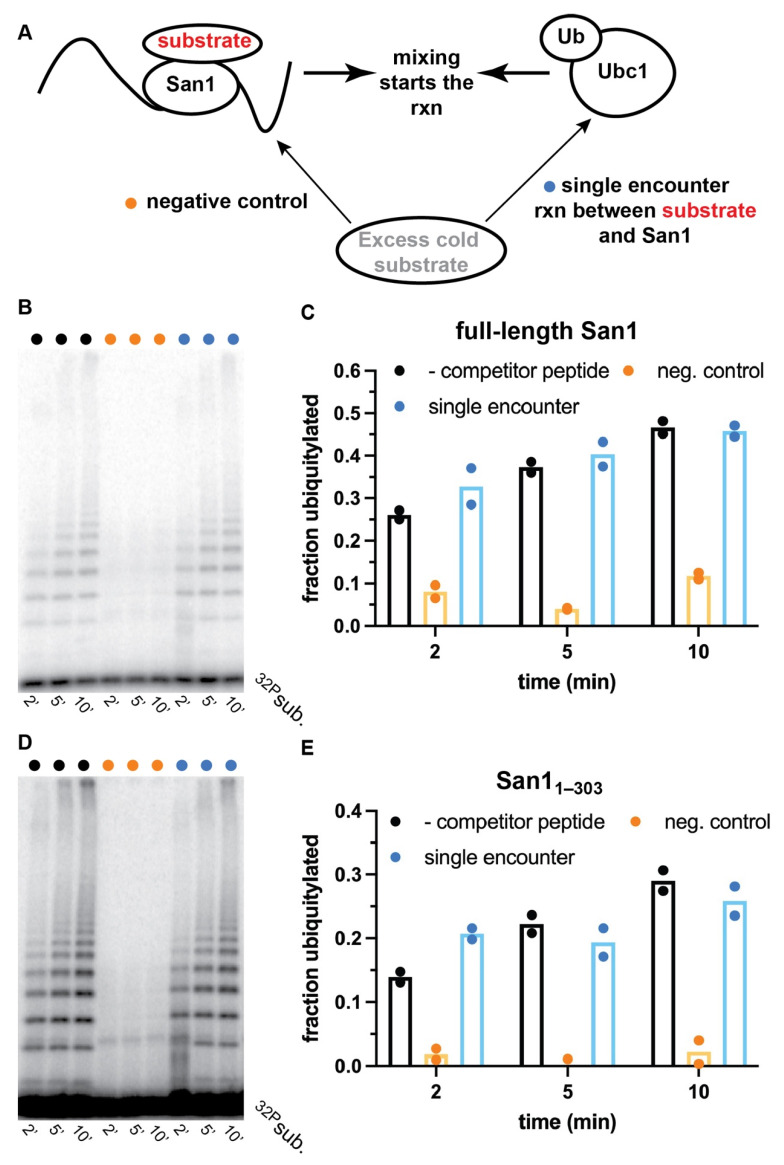
Radiolabeled peptide substrate dissociation from San1 is negligible during single-encounter ubiquitylation reactions. (**A**) Schematic showing how ubiquitylation reactions that are single-encounter for San1 and substrate are assembled. (**B**) Representative autoradiogram of ubiquitylation reactions between radiolabeled peptide substrate and full-length San1. Lanes 1–3 represent time points for a ubiquitylation reaction without excess cold peptide (black dots). Lanes 4–6 represent a negative control reaction where excess cold peptide is first incubated with San1 prior to the addition of radiolabeled substrate (orange dots), and lanes 7–9 represent the results for the single-encounter reaction (blue dots). (**C**) graphical representation of the results in (**B**). (**D**) same as (**B**) except with San1_1–303_. (**E**) same as (**C**), except with San1_1–303_. The results show duplicate data points from technical experimental replicates.

To distinguish between dynamic substrate binding with San1 or the existence of multiple binding sites, ubiquitylation reactions that were single-encounter between substrate and San1 were performed with both full-length San1 and San1_1–303_. A single encounter between substrate and San1 is achieved by first incubating radiolabeled peptide substrate with San1 to form the enzyme-substrate complex, followed by the addition of a solution containing ubiquitin and various enzymes that activate it for transfer to substrate. Excess unlabeled peptide substrate is then added to the activated ubiquitin solution prior to initiation of the ubiquitylation reaction that should outcompete radiolabeled substrate and products that dissociate from San1 (Figure 4A). Ubiquitylation reactions were first performed in the absence of unlabeled competitor substrate, resulting in robust ubiquitylation of radiolabeled peptide substrate (Figure 4B, lanes 1–3, and Figure 4C).

Negative control experiments were then performed with full-length San1 that had been pre-incubated with excess unlabeled peptide prior to the addition of radiolabeled substrate. Almost no substrate ubiquitylation was observed, demonstrating the ability of unlabeled peptide to outcompete radiolabeled substrate (Figure 4B, lanes 4–6, and Figure 4C). Remarkably, in the single-encounter reaction, the fraction of substrate converted to ubiquitylated products was nearly identical in comparison with the positive control reaction at all three time-points tested (Figure 4B, lanes 7–9, and Figure 4C). Highly similar results were observed for single-encounter reactions between peptide substrate and San1_1–303_ (Figure 4D,E). In summary, these results strongly suggest that the peptide substrate as well as ubiquitylated products remain tightly bound to both full-length San1 and to San1_1–303_ over the duration of the time course.

**Figure 5:**
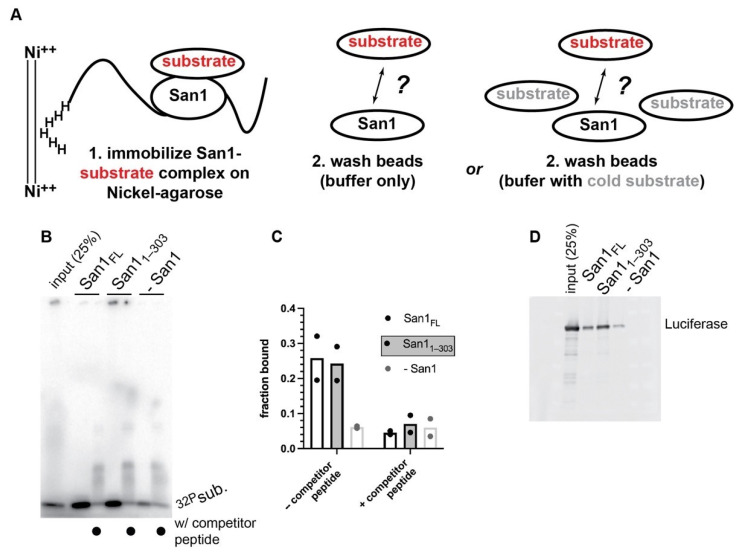
Peptide substrate is bound to San1 with high affinity. (**A**) Schematic showing a nickel-pulldown assay to assess the strength of the San1-peptide substrate complex. (**B**) Representative autoradiogram of the results of the nickel-pulldown assay. (**C**) Graphical representation of the results shown in panel (**B**). The results show duplicate data points from technical experimental replicates. (**D**) same as (**B**) except with heat-denatured luciferase protein substrate.

We next performed direct binding experiments between San1 and substrate. Here both full-length San1 and San1_1–303_ were first incubated with radiolabeled peptide substrate and subsequently immobilized onto nickel-agarose beads followed by washing (Figure 5A). Despite a two hour incubation period with wash buffer, radiolabeled substrate remained bound to both full length San1 and San1_1–303_ (Figure 5B,C). However, the addition of excess unlabeled substrate peptide to the wash buffer resulted in substantial dissociation of labeled substrate from bead-bound San1 to levels comparable with negative control pull-downs that lacked San1 (Figure 5B,C). Nickel pull-downs with full-length San1 or San1_1–303_ and heat-denatured luciferase substrate also resulted in tight binding that was highly resistant to stringent washing conditions, demonstrating that San1 binds to both a small peptide as well as a misfolded globular protein with high affinity.

### 3.2. San1 Substrate Binding Sites Display Specificity

**Figure 6:**
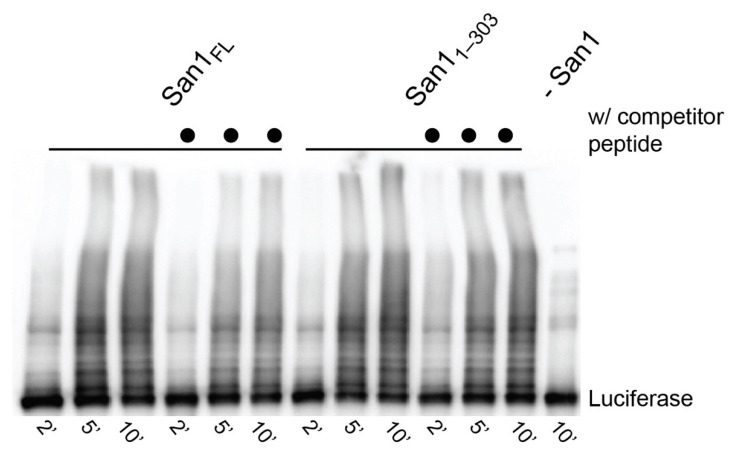
San1 substrate binding sites display specificity. Multi-turnover ubiquitylation reactions between full-length San1 or San_1–303_ and heat-denatured luciferase substrate. To assess substrate specificity, San1 was pre-incubated with unlabeled KR peptide substrate prior to the addition of luciferase (lanes 4–6 and 9–12, San1 or San_1–303_, respectively).

Having established that San1 forms a tight complex with substrates, we next addressed whether San1 substrate binding sites display substrate specificity. Ubiquitylation reactions were performed with heat-denatured luciferase substrate and in the presence or absence of competitor peptide substrate (KR San1 peptide). Full-length San1 or San1_1–303_ were pre-incubated with unlabeled competitor peptide substrate followed by the addition of heat-denatured luciferase and activated ubiquitin. Surprisingly, luciferase was strongly ubiquitylated despite the presence of competitor peptide (Figure 6). Indeed, the fraction of luciferase converted to ubiquitylated products was comparable with positive control reactions lacking competitor peptide. These results strongly contrast with the single-encounter reactions (Figure 4), supporting the notion of San1 having multiple substrate binding sites that have the capacity to display specificity.

## 4. Discussion

It had been known for some time that San1 contains multiple disordered regions, and their systematic deletion in yeast led to defects in both substrate binding and degradation. Our goal was to characterize San1 substrate binding in vitro using direct experimental approaches including biochemical and enzymological assays. While experiments were performed with full-length San1, the presence of several degradation products in that sample made unambiguous interpretation of the results challenging. As such, the same experiments were also performed with San1_1–303_, a C-terminal truncation that enabled far greater levels of purity in comparison with full-length San1, and encouragingly led to nearly identical results as with full-length. The results are all consistent with a model where San1 binds to misfolded substrates through the action of multiple binding regions that have distinct affinities for unique substrates.

An intriguing observation from the kinetic experiments is that the fraction of peptide substrate converted to ubiquitylated product was consistent for both full-length San1 and San1_1–303_ over a very broad range of substrate concentrations (Figure 3). Indeed, nearly 50% of substrate was converted by full-length San1 to product, suggesting that, on average some nine substrate peptides were bound to San1 at the highest ratio of substrate to ubiquitin ligase (18:1). However, only 15% of substrate was converted to product with San1_1–303_ over the same incubation period and the same substrate to ligase ratio. What can be gleaned from these observations regarding San1′s mechanism? In our model, when substrate levels are low, only the highest affinity binding sites are occupied to promote substrate ubiquitylation. The titration of substrate concentrations to higher levels leads to additional sites being occupied until eventually saturation is achieved. However, note that saturation was not achieved here even at the maximal substrate to San1 ratio (18:1) for full-length San1 or for San1_1–303_. The model also suggests why San1_1–303_ converts a lower fraction of substrate to product than full-length. The truncation of the C-terminus to the RING domain seems to have deleted at least some of the binding sites for the peptide substrate, although alternative explanations cannot be ruled out since the peptide solubility is insufficient to achieve saturation of either San1 protein.

Our model also explains why San1 appears to have such remarkable affinity for substrates. The existence of multiple substrate binding sites along a disordered polypeptide chain is likely to result in their achieving proximity with each other. The random coalescing of these binding sites is conceptually similar to the principle of how other avidity-dependent binding events such as affinity chromatography occur. San1 shows avid binding to substrate since the dissociation of a substrate from San1 would likely result in rapid rebinding owing to the high local concentration of unoccupied binding sites. Additionally, similar to affinity chromatography, one means for disrupting the San1-substrate complex is through the addition of excess substrate which was observed during the nickel pull-down binding studies reported here.

Our results stand in striking contrast with how more typical ubiquitin ligases create binding energy with their substrates. For instance, the cullin-RING ligases are the largest and perhaps best characterized family of ubiquitin ligases to date [48]. Here, substrate specificity and affinity are afforded by highly specific inter-molecular interactions between ubiquitin ligase and substrate, leading to sub-micromolar equilibrium dissociation constants. However, consider that the half-life of a typical cullin-RING ligase-substrate complex is only a few seconds [49], in contrast with the results presented here where the half-life is orders of magnitude longer. Under this scenario where the cullin-RING ligase-substrate complex is fleeting, a minor but significant fraction of substrate will dissociate from the ubiquitin ligase prior to its ubiquitylation. Thus, these aborted complexes need at least one more round of substrate binding to the ubiquitin ligase for a chance at achieving ubiquitylation. For the cullin-RING ligases, this may delay ubiquitylation by seconds. However, due to the aggregation-prone nature of PQC substrates, even their fleeting existence in the cell may be detrimental to its survival. Thus, PQC ubiquitin ligases may act like molecular fly paper, binding very tightly to their substrates to prevent their aggregation. Interestingly, it has been shown that some PQC substrates rely on a AAA ATPase called Cdc48 (p97 in humans) to promote ubiquitylation and/or degradation [31,36]. It is tempting to speculate that Cdc48 dependency may also be important to help dissociate tightly bound ubiquitylated substrates from San1.

Like all investigations, our results point to additional experiments that are necessary to further unravel the mechanism of how PQC ubiquitin ligases recognize and bind to misfolded protein substrates. Nevertheless, the results demonstrate how a PQC-specific ubiquitin ligase has evolved an apparently unique mechanism in comparison with more typical ligases for establishing tight substrate binding, and we sincerely hope that these results will embolden other researchers in the field to further develop quantitative in vitro assays for San1 as well as for their PQC-specific ubiquitin ligases of interest. It is also our hope that the demonstration that San1_1–303_ is possibly amenable to sophisticated in vitro experiments will lead to more quantitative assays as well as perhaps structural biological efforts, as we consider these prerequisite before a detailed molecular understanding of PQC ubiquitylation can be achieved.

## Data Availability

Not applicable.

## Supplementary Materials

The following are available online at https://www.mdpi.com/article/10.3390/biom11111619/s1, Figure S1: Peptide substrate significantly protects both full-length San1 and San1_1–303_ from proteolysis.
