# Supplementary material for: The San1 Ubiquitin Ligase Avidly Recognizes Misfolded Proteins through Multiple Substrate Binding Sites
Source: Biomolecules. 2021 Nov 2;11(11):1619. doi: 10.3390/biom11111619 (PMC8615460; doi:10.3390/biom11111619)
Supplement: Supplementary file 1 [file biomolecules-11-01619-s001.zip › biomolecules-1392881-supplementary.pdf]

biomolecules

Supplementary Material

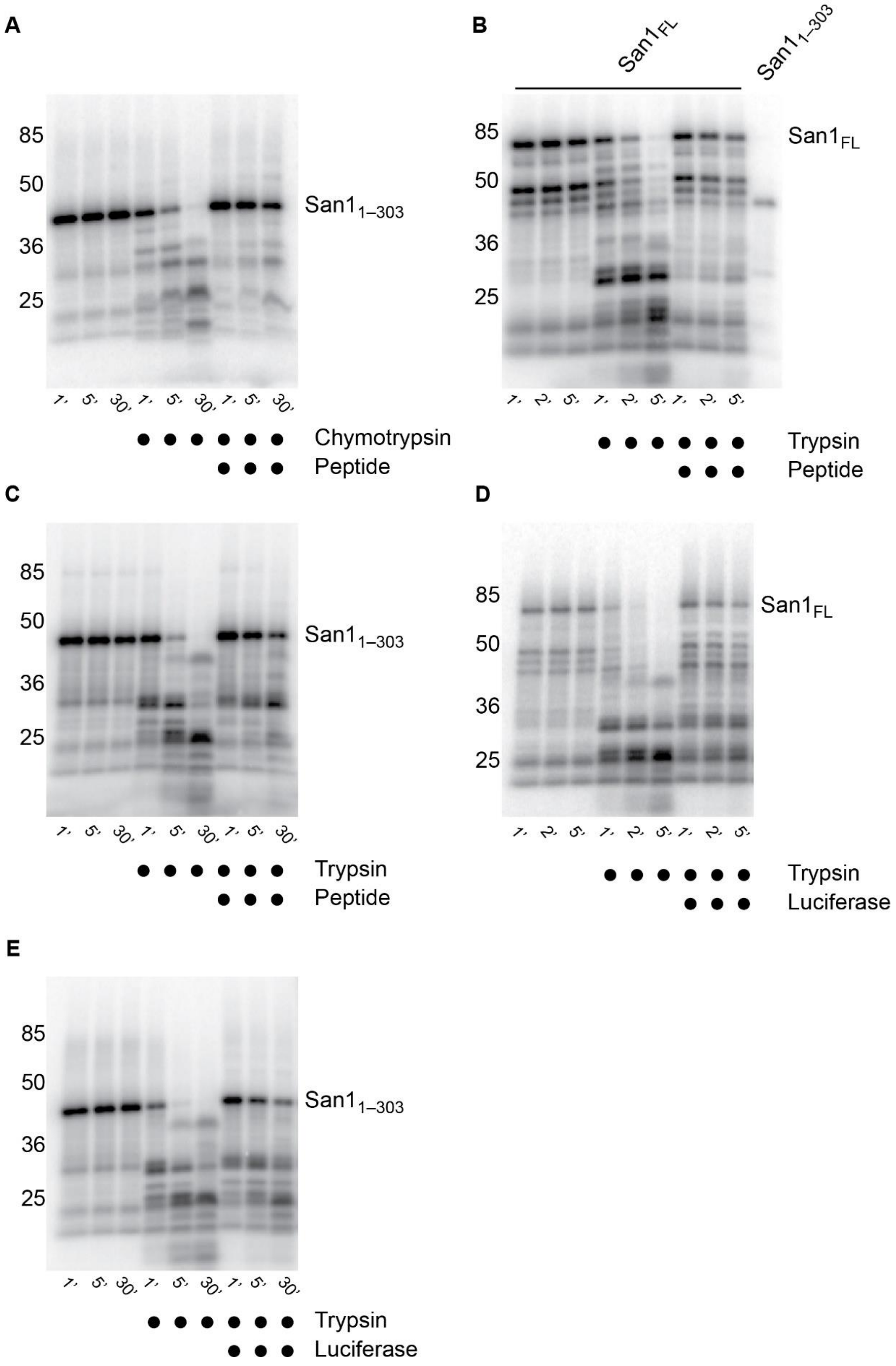

Figure S1. Peptide substrate significantly protects both full-length San1 and San1$_{1-303}$ from proteolysis.
